# Perspective: Infant Feeding Policies among Women Living with HIV in Latin America and the Caribbean: Should They Be Updated?

**DOI:** 10.1016/j.advnut.2025.100469

**Published:** 2025-06-24

**Authors:** Rafael Pérez-Escamilla, Sonia Hernández-Cordero, Tarini Gupta

**Affiliations:** 1 Department of Social and Behavioral Sciences, Yale School of Public Health, New Haven, CT, United States; 2 Research Center for Equitable Development EQUIDE, Universidad Iberoamericana, Mexico City, Mexico

**Keywords:** breastfeeding, HIV, AIDS, mother-to-child-transmission, highly active antiretroviral treatment (HAART), Latin America and the Caribbean, infant feeding policy, women living with HIV

## Abstract

Among women living with HIV (WLHIV), adherence to highly active antiretroviral treatment (HAART) combined with undetectable maternal viral loads and exclusive breastfeeding during the first 6 mo of life dramatically reduces the risk of mother-to-child transmission of HIV. This knowledge has led to updated World Health Organization infant feeding guidelines for WLHIV, calling for governments to support safe breastfeeding practices among WLHIV who want to breastfeed their infants by providing universal access to HAART, viral load tracking, and high-quality breastfeeding counseling and other needed support across settings. These guidelines eventually led several high-income countries, including the United States and Canada, to revise their infant feeding guidelines that previously contraindicated breastfeeding among WLHIV to incorporate safe, evidence-based breastfeeding recommendations for WLHIV. However, in most of the rest of the Americas, breastfeeding contraindication remains in place. We strongly recommend that all countries in Latin America and the Caribbean consider updating their breastfeeding guidance for WLHIV to allow for safe breastfeeding. Implementing the updated evidence-based recommendations poses major implementation challenges as there is no room for error. Systems-driven implementation science research will be needed to understand how best to codesign, implement, scale up, and sustain intersectoral and equitable person and family-centered policies and programs to empower WLHIV to breastfeed safely if they have the choice to do so.

**Statement of significance**

The advent of highly active antiretroviral treatment (HAART) in 2010 became a game changer that led to revised recommendations on infant feeding practices among women living with HIV (WLHIV). Indeed, when WLHIV are adherent to HAART, have an undetectable viral load, and breastfeed exclusively, the risk of mother-to-child transmission by any route becomes exceedingly small. In this perspective, we provide the evidence supporting this statement and why it should continue informing updates in infant feeding policies and program implementation among WLHIV across world regions.

## Introduction

### Mother-to-child transmission of HIV via breastfeeding: discovery phase

The first cases in the United States of men with HIV were reported in 1981, among infants in 1982, and among women in 1983 [1]. By the late 1990s, it became clear in Africa that the transmission of HIV would happen predominantly through heterosexual relationships and that the pandemic could eventually become more prevalent among women than men [2].

The evidence for human milk transmission initially came mainly from epidemiological studies, subsequently confirmed with laboratory studies based on viral isolations and detection of anti-HIV antibodies in breast milk. Retrospective case studies showed that breastfed children whose mothers were infected postpartum with contaminated blood transfusions, and prospective studies found that mother–infant pairs seroconverted postpartum [3].

In 1985, *The Lancet* published the first report of the infection of an Australian breastfed infant, after the mother becoming infected with HIV as a result of a contaminated blood transfusion [4]. The same year, Belgian scientists isolated the virus causing AIDS, from breast milk samples provided by 3 women from sub-Saharan Africa [5]. In 1988, HIV was detected in breastmilk by electron microscopy [3]. Thus, it became evident that the vertical mother-to-child transmission (MTCT) through breastfeeding was a global public health threat.

Studies conducted between 1991 and 1994 estimated that the risk of MTCT among ever-breastfed compared with formula-fed infants was as high as 46% but confidence intervals were very large [6]. These studies also documented that the high viral load in colostrum or early breast milk increased MTCT risk during the early stages of lactation [7]. Research conducted in Malawi between 1994 and 1997 indicated that MTCT among women living with HIV (WLHIV) who breastfed for 2 y was ≥10.3%. A trial conducted in Kenya during 1992–1998 that randomly assigned women to breastfeed or formula feed showed that *1*) the MTCT via breastfeeding represented an additional 16.2% risk beyond the risk of transmission in-utero or during labor and delivery, *2*) the highest risk of transmission happened during early breastfeeding, and *3*) that formula feeding prevented 44% of MTCT and significantly improved infant’s HIV-free survival [8]. De Cock et al. found in a meta-analysis of 5 clinical trials published in 2000 that was subsequently confirmed by the World Health Organization, that the risk of MTCT via breastfeeding was indeed substantial (Table 1) [9,10].

**TABLE 1:** Risk of mother-to-child-transmission (MTCT) among untreated women living with HIV.

MTCT period	Risk (%)
During pregnancy	5–10
During labor and delivery	10–15
Without breastfeeding	15–25
Breastfeeding to 6 mo postpartum	20–35
Breastfeeding to 18–24 mo postpartum	30–45

Based on analyes by the World Health Organization [9] and De Cock et al. [10].

These estimates were consistent with those from Piwoz and Ross [11]. The advent of highly active antiretroviral treatment (HAART) became a game changer as it dramatically reduced the risks of MTCT via breastfeeding, in-utero, and perinatally. Indeed, when WLHIV are adherent to HAART, have an undetectable viral load, and breastfeed exclusively, the risk of MTCT by any route becomes exceedingly small [12].

The first objective of this paper is to review the evidence on the epidemiology of HIV MTCT, infant feeding practices among WLHIV, how MTCT transmission can be substantially reduced via HAART, viral load monitoring to confirm undetectable loads, and high-quality and timely breastfeeding support for WLHIV. The second objective is to describe how this knowledge transformed the WHO infant feeding guidelines for WLHIV in such a way that, under certain conditions, it is possible for WLHIV to safely breastfeed regardless of the level of economic development of the country. The third objective is to present and discuss challenges for effectively implementing such recommendations in the real world. The fourth objective is to identify research gaps that will need to be filled with future research designed to understand how to effectively implement the most recent guidance under very contrasting economic, social, political, and healthcare systems contexts around the world. Our focus is on Latin America and the Caribbean (LAC), given that almost all countries in this region still contraindicate breastfeeding among WLHIV, whereas in the Americas, the United States, and Canada have already updated their recommendations. For these reasons, we present the situation in Latin America, the United States, and Canada in different sections. We expect this paper to provide evidence that decision makers in LAC need to decide whether they want to move forward or not with consultations that may lead to updating the infant feeding recommendations among WLHIV.

### Epidemiology of maternal and pediatric HIV

The HIV epidemic remains a significant global challenge. In 2023, 39.9 million (range: 36.1–44.6 million) adults and children were living with HIV worldwide. Among new daily cases, 3200 of 3600 were among people aged 15 y and older, with 44% occurring in women.

Sub-Saharan Africa experiences 65% of the global HIV burden, and LAC and Western and Central Europe and North American regions have a prevalence of 5.8% each [13]. Importantly, LAC is 1 of the 3 regions experiencing rising new HIV infections [13]. The majority of the world's HIV infections are among women with 51.4% of the cases being detected among women aged 15 y or older [14]. In LAC, ∼2.3 million people (range: 2.1 million–2.6 million) were living with HIV in 2023 [15] (Table 2).

**TABLE 2:** Regional estimates of the number of adults and children living with HIV by 2023.

Region	Adults and childrenliving with HIV
Eastern and Southern Africa (million)	20.8
Western and Central Africa (million)	5.1
Middle East and North Africa (thousands)	210
Asia and the Pacific (million)	6.7
Latin America (million)	2.3
Caribbean (thousands)	340
Eastern Europe and Central Asia (million)	2.1
Western and Central Europe andNorth America (million)	2.3
GLOBAL (million)	39.9

UNAIDS, 2024 [14].

### In utero, intrapartum, and postpartum HIV transmission

MTCT has decreased globally, in 1989, 1 in 4 children born to WLHIV became infected, but currently, ≥15 countries have practically eliminated MTCT. As of 2021, the global MTCT rate was 11.9% [16], by 2023 the estimated mean MTCT in LAC ranged from 3.9% [95% confidence interval (CI): 3.6%, 4.2%] in Chile to 27.6% (26.2%, 29.1%) in Guatemala [15,17]. HAART adherence during pregnancy, labor, and breastfeeding significantly reduces the risk of MTCT. However, structural barriers continue to limit access to HAART for women of reproductive age, preventing adherence, and limiting access to other pre-exposure prophylaxis options. In 2023, 84% (95% CI: 72%, 98%) of pregnant WLHIV had access to antiretrovirals (ARV) to prevent MTCT, which fell short of the global goal for 2025 of universal access to HAART for all pregnant WLHIV [13]. Noticeably, in LAC, only 65% (57%, 77%) of pregnant WLHIV are receiving HAART [18].

Breastfeeding is the best way to nourish newborns and young children as it reduces the risk of mortality and morbidity from all causes, has a positive lasting impact on their development, and reduces the risk of obesity [19]. Previously, WLHIV, especially in middle-upper and high-income countries, were advised not to breastfeed in the context of guaranteed access to safe infant feeding replacement alternatives. Considering the new evidence on the strong protection of HAART against MTCT, some of these countries, including the United States, but not all, have now modified the breastfeeding contraindication to allow WLHIV who want to breastfeed to be able to do so safely when certain conditions are met.

### Infant feeding practices among WLHIV

WLHIV are less likely to express the intention to breastfeed compared with women not living with HIV. A study from South Africa found that women not living with HIV were more likely to plan breastfeeding compared with WLHIV (98.9% compared with 70.6%, respectively, *P* < 0.001) [20]. Evidence from countries with high HIV prevalence, including South Africa, indicates that some WLHIV have chosen to breastfeed despite medical advice to the contrary. This decision was often influenced by HIV-related stigma, as formula feeding could be perceived as an indication of being HIV-positive, leading to social disapproval [21]. Overall, it has been documented that WLHIV are less likely to breastfeed at any point, and few continue breastfeeding for >6 mo [20,22,23].

In the United States and other high-income countries [24], a significant proportion of WLHIV breastfeed in spite of being strongly counseled against doing so.

### Evolution of the WHO infant feeding recommendations for WLHIV in the context of HAART

The WHO guidelines for infant feeding among WLHIV have evolved in tandem with the advent and scaling up of HAART. HAART dramatically reduces MTCT risk. As a result, the WHO infant feeding guidelines for WLHIV strongly evolved between 1998 and 2016 from contraindicating or strongly advising against breastfeeding when safe infant feeding replacements were available, to recognizing that safe breastfeeding among WLHIV was possible when certain conditions were met, including access and adherence to HAART.

### 1988 WHO guideline

The first WHO infant feeding guideline for WLHIV identified longer breastfeeding duration as a risk factor for MTCT [25]. Strategies to reduce MTCT emphasized the need to improve access to HIV testing and provide women with information about HIV and MTCT, in addition to expanding access to condoms and safe blood transfusions. Since then, to reduce MTCT among WLHIV, it was recommended for them to have access to antiretroviral treatment (ART), safe replacement feeding, and noninvasive obstetric procedures.

Unlike the guidelines updated in 2010 and 2016, this guideline acknowledged that although breastfeeding is the best way to feed an infant, replacement feeding was recommended for WLHIV to reduce the risk of infant mortality from HIV, if they had access to acceptable, feasible, affordable, sustainable, and safe (AFASS) replacement feeding options [26]. Voluntary counseling and testing was emphasized, including pretesting counseling by healthcare professionals providing education on HIV testing, routes of MTCT, and implications of different infant feeding decisions for MTCT risks [27]. For WLHIV who chose to breastfeed, early cessation of breastfeeding was recommended to address the increased risk of MTCT with increased breastfeeding duration.

The guideline also recommended that WLHIV stop breastfeeding as soon as they had access to AFASS replacement feeding options. At the same time, it urged health systems to promote breastfeeding for non-HIV-infected mothers and adhere to the International Code of Marketing of Breastmilk Substitutes and implement the Baby Friendly Hospital Initiative. Overall, the 1998 WHO recommendations were of limited value given the lack of access to HIV testing, ARTs, and AFASS replacement feeding options in the regions of the world where the vast majority of MTCT was happening, especially sub-Saharan Africa [28]. Soon after the 1988 recommendations were published, they were updated in 2001 to reflect evidence that had emerged [26]. The updated guidance emphasized the need for breastfeeding cessation as soon as AFASS replacement feeding became available, along with providing frequent counseling to help mothers navigate this transition while protecting maternal breast health and ensuring optimal infant nutrition.

Table 3 presents a comparison of key characteristics of the WHO Infant Feeding Guidelines for WLHIV over time.

**TABLE 3:** Comparison of WHO infant feeding guidelines for women living with HIV^1^.

Topic	1998	2010	2016
EBF recommendation and continued breastfeeding	EBF not recommended, instead it recommended replacement feeding from birth with breastmilk substitutes	In settings promoting breastfeeding with ARV use, mothers living with HIV should exclusively breasted for 6 mo, continue breastfeeding ≤12 mo with complementary feeding, and stop only when a safe, adequate diet without breast milk is available	Exclusive breastfeeding for 6 mo, continued for ≥12 mo, and ≤24 mo or beyond with full ART adherence support
ART integration	Recommended for mothers with known HIV status	Maternal ART introduced for breastfeeding	Lifelong ART for all mothers
Biological evidence	Focus on non-HIV mortality risks	ART reduces viral load; evidence from trials	ART minimizes residual transmission risks
Acceptable, feasible, affordable, sustainable, and safe (AFASS) replacement feeding	Recommended for mothers with known HIV status for the first 6 mo. After this, replacement feeding should continue to include an appropriate breast milk substitute and complementary foods	Simplified AFASS replacement feeding guidance	Allowed with ART if breastfeeding not feasible
Mixed feeding guidance	Discouraged	Limited guidance	Permitted with ART when necessary
Health systems	Train all health workers on HIV and infant feeding counseling		National or subnational health authorities should decide whether health services will principally counsel mothers known to be HIV-infected to either breastfeed and take antiretrovirals, or, avoid all breastfeeding
Voluntary counseling and testing (VCT)	Counseling and testing recommended with consent and confidentiality		Shift toward a public health approach
ART for baby	Infant antiretroviral prophylaxis was not recommended	A short course of infant prophylaxis (typically nevirapine for 6 wk) was recommended to reduce postnatal HIV transmission.	Infant prophylaxis (for example, 6 wk of nevirapine) remained recommended as an integral component of the MTCT package, even as maternal lifelong ART further minimizes transmission risk.

Abbreviations: ART, antiretroviral treatment; EBF, exclusive breastfeeding; MTCT, maternal-to-child transmission.

1 The 1998 guidelines were primarily based on expert opinion with limited empirical data. The 2010 recommendations were based on systematic reviews and the Grading of Recommendations Assessment, Development and Evaluation (GRADE) system, integrating clinical study data. The 2016 update further refined these recommendations using additional systematic reviews and modelling, with evidence quality assessed across outcomes, and input from implementors.

### The advent of combined ART in the context of exclusive breastfeeding

Following the evidence published since 1999 that exclusive breastfeeding significantly reduced the risk of MTCT in South Africa and Zimbabwe [29,30], the demonstration that HAART combined with exclusive breastfeeding for the first 6 mo after birth dramatically reduced the risk of MTCT transmission became a game changer. The data came mainly from sub-Saharan Africa, as at the time, the policy in middle and high-income countries was to contraindicate breastfeeding for WLHIV; however, because of its strong biological plausibility, high-income countries also started to reconsider their contraindication for breastfeeding among WLHIV [24].

As reported by Morrison [31], the seminal Mma Bana study conducted in Botswana by Shapiro et al. [32] showed that among WLHIV who exclusively breastfed for 6 mo and had received HAART during pregnancy and after birth, only 0.28% (2/709) of infants became infected. The 2 cases of MTCT were explained by a lack of adherence to HAART or having a detectable viral load while breastfeeding. Studies conducted in Tanzania, Mozambique, Rwanda, Uganda, and Zambia with mothers who were exclusive breastfeeding and provided with HAART during pregnancy and breastfeeding confirmed these findings, that is, the risk of MTCT when exclusive breastfeeding was combined with HAART adherence was <1%. The risk ranged from 0% (0/184) in rural Tanzania when maternal viral load was 100–1000 copies/mL among mothers who breastfed exclusively for 6 mo, followed by continuing breastfeeding until 12 mo postpartum once complementary foods were introduced [33]. These mothers received HAART throughout pregnancy, and their infants received daily nevirapine for 4–6 wk after birth. In this study, MTCT occurred in 2 of 186 infants, 1 with a mother with high viral loads 5 wk after birth, and another one with a mother that interrupted HAART while continuing to breastfeed. At the other end of the range, MTCT risk in Zambia was 1.5% (3/201) at 12 mo postpartum [34], which was unexpected given that the mothers in that study also received HAART during gestation, labor, and delivery and until they stopped breastfeeding and their infants received zidovudine for 5 d postpartum. No MTCTs occurred among infants being exclusively breastfed. All MTCTs happened between 6 and 12 mo postpartum among mothers with high viral loads due to poor HAART adherence, as expected.

The Kesho Bora study conducted in 2005–2008 in Burkina Faso, Kenya, and South Africa raised concerns as it found an MTCT risk through breastfeeding among women receiving HAART ranging from 2.1% at 6 wk to 4.5% at 12 mo postpartum [35]. Thomas et al. [36] took a close look at this study and found that this happened because only 70% of women in the study had undetectable viral loads at delivery, and follow-up postpartum tracking of viral loads was suboptimal. In addition, only 45% of infants were breastfed exclusively before 3 mo postpartum. In agreement with these findings in lower-income countries, studies in North America and the United Kingdom report no cases of vertical transmissions among breastfeeding mothers with an undetectable viral load [[37], [38], [39]].

An important finding from the HAART body of evidence that has strong implementation implications for MTCT prevention programs is that the efficacy of HAART at suppressing the viral load to undetectable levels is a function of the maternal viral load at the start of treatment [31]. Hence, to prevent MTCT, it is key to start HAART as early as possible in pregnancy, which requires access to universal access to timely prenatal care. Lastly, the impact of HAART at preventing MTCT among infants who are mixed fed (that is, being fed both breast milk and other milks, usually commercial milk formula) during the first 6 mo of life remains to be determined.

In summary, the evidence shows that WLHIV who want to breastfeed need to have access to timely HAART, viral load tracking, and support for exclusive breastfeeding to prevent MTCT.

The following sections present how the HAART-MTCT discoveries led to major infant feeding WHO recommendation changes for WLHIV as reflected in the 2010 guideline and the 2016 implementation guidance.

### 2010 WHO guideline

The 2010 guideline promoted HAART access in combination with exclusive breastfeeding for 6 mo and complementary feeding starting at this time [40]. These recommendations were also more in line with a growing public health concern of recommendations calling for AFASS infant feeding replacements as the primary option.

For the first time, the WHO explicitly recommended ARV drug interventions to prevent MTCT through breastfeeding. Mothers on lifelong ART or those receiving prophylactic ARVs were advised to exclusively breastfeed for 6 mo, introduce complementary foods thereafter, and continue breastfeeding for ≤12 mo, stopping only when a nutritionally adequate and safe replacement alternative became available. The guideline emphasized that ARV therapy significantly reduced postnatal MTCT risk to almost negligible and aimed to balance the risk of HIV transmission with the increased mortality risk from diarrhea, pneumonia, and malnutrition associated with unsafe formula feeding, ultimately prioritizing HIV-free survival.

The evidence supporting these recommendations highlighted the protective role of exclusive breastfeeding compared with mixed feeding, which carried a higher risk of HIV transmission and other morbidities. Modeling studies commissioned for the guideline suggested that breastfeeding with ART interventions provided the highest HIV-free survival time, calling for the provision of skilled counseling and support in appropriate infant feeding practices and ARV interventions. In settings where national or subnational authorities endorsed breastfeeding with ART as the primary feeding strategy, the guidelines strongly recommended exclusive breastfeeding for the first 6 mo, followed by complementary feeding while continuing breastfeeding ≤12 mo. The 6-mo exclusive breastfeeding recommendation was based on high-quality evidence, whereas the guidance for continued breastfeeding ≤12 mo was supported by lower-quality evidence.

This guideline also addressed cases where WLHIV chose to stop breastfeeding, discouraging abrupt weaning as previously recommended in the 1998 guidelines. Additionally, if either the mother or infant was receiving ART or ARV prophylaxis, it was advised to continue treatment for 1 wk after breastfeeding fully stopped to further reduce the risk of postnatal HIV transmission. The guideline also recommended that, in specific circumstances such as when an infant was born with low birth weight, when the mother was temporarily unwell, or if ART was temporarily unavailable, mothers should consider expressing and heat-treating breast milk as an interim feeding strategy to ensure a safer alternative to direct breastfeeding.

In sum, the 2010 guideline shifted responsibility from the WLHIV to the governments to support their ability to safely breastfeed their infants by providing universal access to ART and qualified breastfeeding counseling and support.

### 2016 WHO implementation guidance

The 2016 WHO guidance on HIV and infant feeding expanded on the 2010 recommendations by extending the recommended duration of breastfeeding and reinforcing the importance of lifelong ART for all HIV-positive mothers [40]. The guidance development process was robust, including systematic reviews, benefit-risk modeling studies, and allowing extensive input from policy makers and implementors, taking equity considerations into account. The updated guidance recommended that WLHIV should exclusively breastfeed for the first 6 mo, introduce appropriate complementary foods thereafter, and continue breastfeeding for ≥12 mo. It reiterated that breastfeeding should only cease once a nutritionally adequate and safe diet without breast milk could be ensured. This recommendation was based on a consensus within the Guideline Development Group, which determined that the likely benefits outweighed potential harm. Although acknowledging that the protective effect of breastfeeding against serious morbidity and mortality is stronger in the first year of life than in the second, the Guideline Development Group recognized that in settings where health systems effectively support retention in care and adherence to ART, the risk of postnatal HIV MTCT was likely to be low. As ART programs became more widely implemented and accepted, the protective benefits of prolonged breastfeeding with ART provision were expected to strengthen. Additionally, aligning infant feeding recommendations for WLHIV with those for the general population was seen as a critical step in reducing stigma associated with non-normative feeding practices and improving overall infant feeding practices.

A key focus of the 2016 guidance was calling for strong support for optimal infant feeding for WLHIV by national health authorities. It emphasized the need for skilled counseling and structured support to promote exclusive and continued breastfeeding alongside ART adherence at the facility and community level. Addressing barriers to sustained breastfeeding, such as maternal return to work or school, was recognized as essential for program effectiveness. National health authorities were advised to integrate skilled infant feeding counseling and ART support into routine maternal and child health services. The guidance acknowledged that effective implementation required coordination between healthcare providers, policymakers, and community organizations to ensure consistent messaging and practical support for mothers considering different infant feeding decisions.

The guidance also indicated that ART reduced the risk of postnatal MTCT also for mixed feeding. Although exclusive breastfeeding was recommended, mixed feeding was no longer considered as a reason to stop breastfeeding when adhering to ART without detectable viral loads. However, the guidance acknowledged the need for further research on the long-term impact of mixed feeding in combination with ART, particularly in relation to child health outcomes and MTCT dynamics. Lastly, the guidance did not provide explicit recommendations for mothers with undetectable viral loads who wished to continue breastfeeding beyond 12 mo.

### Changes in infant feeding policies among WLHIV in the United States and other high-income countries

Based on the first infant feeding recommendations for WLHIV, the United States contraindicated breastfeeding among WLHIV, regardless of maternal viral load or combined ARV therapy status [41]. This categorical recommendation was based on the potential of MTCT via human milk in the context of access to safe replacement feeding options. It remained in place until 2023, when healthcare providers were advised by the Department of Health and Human Services that they could empower WLHIV to breastfeed safely if that is the infant feeding choice that they preferred, as long as they followed strict breastfeeding safety criteria. This drastic change in recommendation was the result of calls from public health scholars [42] combined with the conclusive evidence that women adhering to HAART therapy without detectable virus levels could safely breastfeed [43,44] plus community desires illustrated by the fact that a significant proportion of WLHIV in the United States and other high income countries were or would have wanted to breastfeed their infants in spite of having been advised during pregnancy not to do so [24,45]. Indeed, studies conducted in the United States found that WLHIV have chosen to breastfeed [39]. Likewise, a United Kingdom survey found that over a third of WLHIV wanted to breastfeed [46] and similar findings have also been documented elsewhere in Europe [24].

In 2019, Gross et al. [42] strongly recommended for the United States to reconsider its categorical recommendation against breastfeeding among WLHIV based on a literature review. They first estimated the risk of HIV transmission from breastfeeding for HIV-exposed United States infants. Then, they compared this risk in the same population of children if they were not breastfed, including excess mortality from sudden infant death syndrome, necrotizing enterocolitis, sepsis, and also considered maternal health. They concluded that absolute contraindication for breastfeeding for WLHIV may not maximize health outcomes and recommended eliminating the categorical recommendation against breastfeeding for combined ART-adherent WLHIV, also taking into account the principles of autonomy, harm reduction, and social justice.

In 2023, the US Department of Health and Human Services (HHS) published the “Update to Clinical Guidelines for Infant Feeding Supports Shared Decision Making: Clarifying Breastfeeding Guidance for People with HIV” [45] that removed the categorical contraindication for breatsfeeding among WHLIV. The new HHS HIV clinical practice guidelines now incorporate breastfeeding options for WLHIV on ARV therapy with sustained undetectable viral load in the blood. The guidelines state that “People with HIV (PWH) who are considering conception, are pregnant, or in the postpartum period should receive evidence-based counseling to support decision making about infant feeding. The updated guidelines note that: A) the risk of postnatal HIV transmission to an infant is zero with the use of safe replacement feeding. Properly prepared formula or pasteurized human donor milk from a milk bank eliminates the risk of HIV transmission to the infant. B) The risk of HIV transmission while breastfeeding is less than 1% (but not zero) for PWH on antiretroviral therapy (ART) with sustained undetectable viral load through pregnancy and postpartum. C) Clinicians should support the choices of people with HIV to breastfeed (if they are virally suppressed) or to formula/replacement feed.” Importantly, they specifically note that “It is inappropriate to engage Child Protective Services (CPS) or similar services in response to infant feeding choices of PWH.” The United States HHS perinatal guidelines state that individuals should “receive patient-centered, evidence-based counselling on infant feeding options” [43], and similar statements are included in the British, Canadian, Swiss, European, and Australasian perinatal guidelines [47].

Consistent with the HHS updated guidelines, the American Academy of Pediatrics recommended in 2024 with the endorsement of the Centers for Disease Control and Promotion, “For people with HIV in the United States, avoidance of breastfeeding is the only infant feeding option with 0% risk of HIV transmission. However, people with HIV may express a desire to breastfeed, and pediatricians should be prepared to offer a family centered, nonjudgmental, harm reduction approach to support people with HIV on ART with sustained viral suppression below 50 copies per mL who desire to breastfeed. Pediatric health care professionals who counsel people with HIV who are not on ART or who are on ART but without viral suppression should recommend against breastfeeding” [48].

Canada and several European countries [24] changed their original infant feeding guidelines among WLHIV before the United States, removing WLHIV as a categorical contraindication for breastfeeding, and as in the United States, allow for them to be supported with their breastfeeding journey if that is their choice [[49], [50], [51], [52], [53], [54]]. The guidelines were updated based on the new ART evidence in the context of documenting that over time, more WLHIV were practicing or planning to breastfeed even when they were told that it was not recommended because of the potential risk of MTCT [[55], [56], [57], [58], [59]]. For example, in the Netherlands, 71% of 82 WLHIV expressed a desire to breastfeed in the future. The 2 most important factors influencing their decision to breastfeed were the low risk of MTCT and the advice by the doctor or nurse practitioner. Among the study participants, 42% expressed their interest in breastfeeding with a transmission risk <1% and more than half of the participants expressed their interest to breastfeed with the additional monitoring needed [58].

**FIGURE 1:**
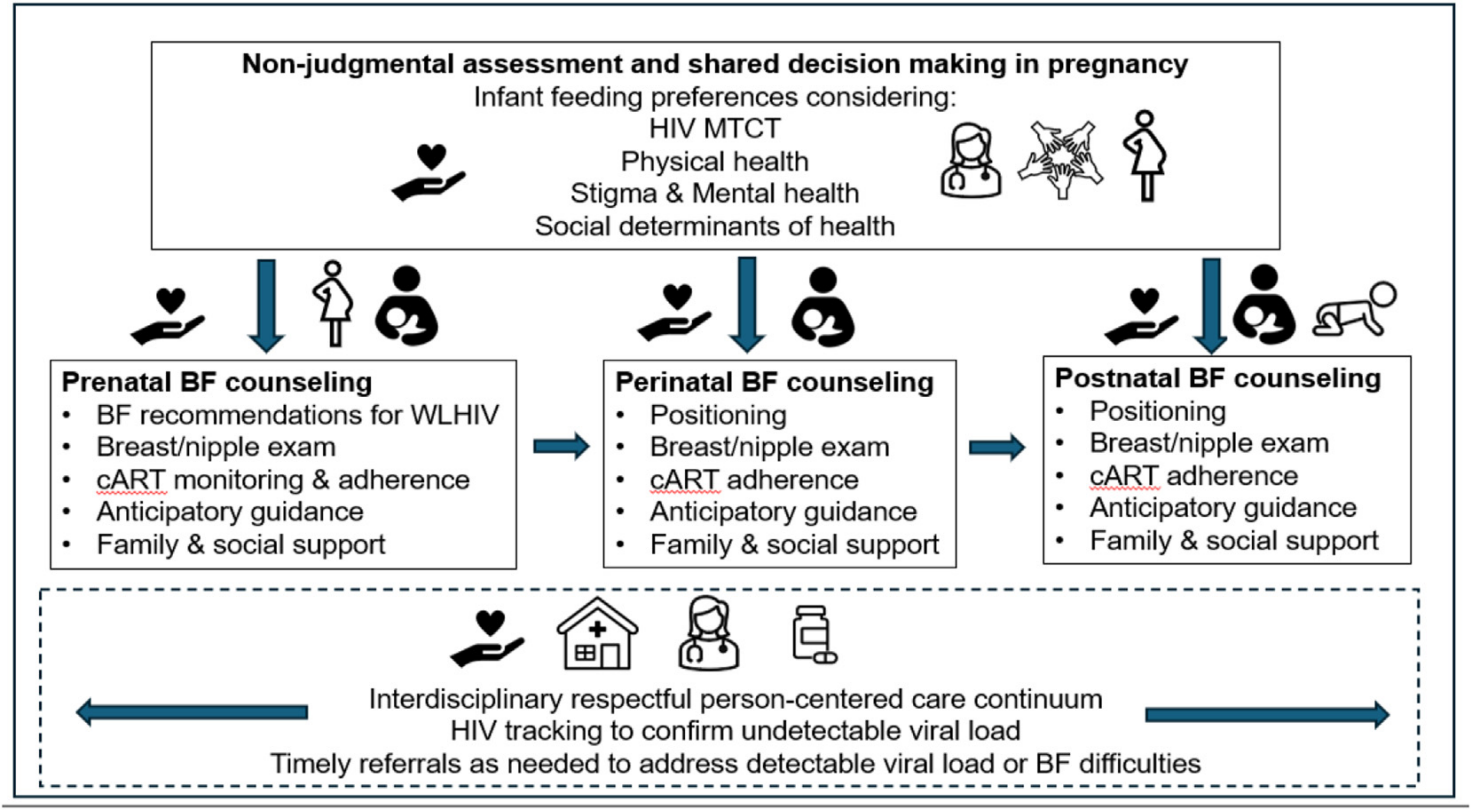
Model for person-centered care for implementation of safe breastfeeding guidance among women living with HIV (WLHIV). BF, breastfeeding; cART, combined antiretroviral treatment; MTCT, mother-to-child transmission.

The optimal care for WLHIV to safely breastfeed has been well established but requires interdisciplinary teams coordinating their work across sectors to provide shared decision making and women-centered care [47,52] (Figure 1).

This is a complex task that poses major implementation challenges involving several interlocked systems with many moving parts and there is little room for error. Hence, research is needed to better understand how to empower WLHIV in the United States and beyond to safely breastfeed their infants [37]. A recent qualitative study conducted in Miami with 20 WLHIV who were recruited at a university Pediatric Infectious Diseases Clinic and interviewed individually in English, Spanish, or Haitian Creole between 2022 and 2023 identified key potential barriers and facilitators for this to happen [60]. Before starting the semistructured interview each WLHIV was told that *1*) to try to make breastfeeding as safe as possible for women with this infection, some conditions are required for the mother: *a*) is fully adherent to antiretroviral therapy (ART); *b*) has had undetectable viral loads throughout pregnancy and maintains undetectable viral loads during breastfeeding; *c*) comes to the clinic and is closely monitored every 1–2 mo. *2*) The infant must *a*) remain on ART while breastfeeding; *b*) be seen in the clinic and get tested often (every 1–2 mo); *c*) is exclusively breastfed (not supplemented with formula). Then, the WLHIV was asked, “Knowing this, would you be interested in breastfeeding your infant, even though you are living with this condition?” Major barriers reported by the WLHIV were related to the interference with daily work and family care activities due to frequency of testing, fear of transmission to the infant via breastfeeding, lack of a standardized approach to breastfeeding education in the context of HIV from healthcare providers, and maternal concerns about administering HIV medications to the infants and peer pressure to breastfeed. Key facilitators included the benefits and advantages of breastmilk, access to more scientific research information on breastfeeding in the context of HIV, advice from a lactation consultant knowledgeable of infant feeding guidelines for WLHIV, emotional connection and attachment with the child as a result of breastfeeding, support from family and partners, empowering and supporting autonomy and decision-making about infant feeding, providing breastfeeding as an additional infant feeding choice, access to the lived experiences of WLHIV who have successfully and safely breastfeed, and collaborative respectful relationships with healthcare providers. Consistent with previous studies [37,47], these findings suggest that WLHIV-centered healthcare and breastfeeding support in the context of shared decision-making through improved healthcare and social services and social support systems will be needed to successfully implement programs that empower WLHIV who wish to breastfeed to do so successfully and safely [47].

A recent participatory formative qualitative study among WLHIV recruited in healthcare facilities in Lusaka, Zambia also concluded that person-centered or tailored counseling and family support for WLHIV is likely to be needed for improving exclusive breastfeeding, child-responsive caregiving, and ART adherence [61].

Although ART uptake among WLHIV is increasing over time, the adherence to HAART remains suboptimal. Data from New York State collected in 2008–2010 found that only 75% of WLHIV were virally suppressed at delivery, and among those, just 44% remained suppressed 1 y postpartum [62]. Retrospective studies from Mississippi and Philadelphia found that >60% of WLHIV were not optimally engaged in HIV care throughout the year after delivery [[63], [64], [65]]. Postpartum nonadherence to ART and loss of care among WLHIV in the United States and beyond remain significant concerns about the safety of breastfeeding for their infants, especially among socioeconomically vulnerable WLHIV.

### Time for change of infant feeding policies among WLHIV in LAC

Through an extensive literature review of legal and policy documents, including national standards, decree, laws, and strategic plans, and consultations with key informants, we were able to examine current infant feeding for WLHIV in 19 countries in LAC. We specifically focused in defining the regulatory and programmatic frameworks for the MTCT of HIV, including infant feeding recommendations for WLHIV. The identified materials were published between 2001 (El Salvador) and 2025 (Argentina and Paraguay). The included countries were Argentina [66,67], Belize [68], Bolivia [69,70], Brazil [71] Chile [72], Costa Rica [73,74], Dominican Republic [75], Ecuador [76], El Salvador [77,78], Guatemala [79,80], Honduras [[81], [82], [83]], Mexico [84,85], Nicaragua [86,87], Panama [88,89], Paraguay [90], Peru [91,92], Trinidad y Tobago [93,94], Uruguay [95,96], and Venezuela [97,98]. Recommendations on infant feeding and on HIV detection and treatment recommendations for preventing MTCT were examined with particular attention to infant feeding guidelines (Table 4) [66,[68], [69], [70], [71], [72], [73], [74], [75], [76], [77], [78], [79], [80], [81], [82], [83], [84], [85], [86], [87], [88], [89], [90], [91], [92], [93], [94], [95], [96], [97], [98]]. Additionally, we described strategies to reduce MTCT, such as early HIV detection in pregnant women, ART during pregnancy and early postpartum, and neonatal prophylactic treatment, included in normative documents across countries in LAC (Supplemental Table 1).

**TABLE 4:** Summary of Infant feeding recommendations among women living with HIV in selected countries from the Latin American Region (2001–2025).

Country	Year	Type of document	Issuing organization	Infant feeding recommendation	Infant feeding support	In charge of its implementation and oversight	Additional notes
1. Argentina [66]^a^	2016	Guideline	Direction of HIV/AIDS and STIs, Ministry of Health of the Nation, UNICEF	In all cases, breastfeeding contraindicated	The provision of formula milk must be ensured, provided by the Directorate of HIV and STIs of the Ministry of Health of the Nation, from the start for all newborns of mothers with HIV, as well as coverage with lactation inhibitors.	Directorate of HIV and STIs of the Ministry of Health of the Nation	Continuation of antiretroviral therapy in women after childbirth is not mentioned in the document. The reason is that the guidelines focus solely on the prevention of HIV transmission to the infant.
2. Belize [68]	2012–2016	National Strategic Plan	Ministry of Health	The National Strategic Plan does not explicitly contraindicate breastfeeding but recommends providing milk substitutes to infants born to HIV-positive mothers.	The Ministry of Health provides free replacement feeding for the first 10 mo to children born to HIV-positive mothers.	Ministry of Health; National AIDS Commission, National AIDS Commission.	Postpartum antiretrovirals for women not mentioned.
3. Bolivia [69,70]	1. 2007 2. 2017	1. Law 2. Guideline	1. Honorable National Congress of Bolivia 2. Ministry of Health of the Plurinational State of Bolivia	HIV-Positive women advised not to breastfeed (Guideline)	Not specified in law or guideline.	The National Sexual Transmitted Infections (STI)/HIV/AIDS Program of the Ministry of Health	Newborns of mothers with HIV must undergo an HIV diagnostic test. Children born to mothers living with HIV-AIDS have the right to receive specialized pediatric services, including access to antiretroviral medications.
4. Brazil [71]	2020	Guideline	Ministry of Health of Brazil, Secretariat of Health Surveillance, Department of Chronic Conditions and Sexually Transmitted Infections	a) The mother should be advised to replace breast milk with infant formula until the child reaches 6 mo of age. b) Cross-nursing (feeding the child by another wet nurse) and the use of pasteurized human milk at home are strictly contraindicated.	The government provides free infant formula for children born to HIV-positive mothers.	The Brazilian Ministry of Health, through the Secretariat of Health Surveillance, in coordination with state and municipal health departments	In Brazil, there has been a law since 1996 that stipulates, “People living with HIV and those with AIDS will receive, free of charge, all necessary medication for their treatment from the Unified Health System.”
5. Chile [72]	2012	National standard	Public Health Under secretariat, Ministry of Health	1. Breastfeeding must be discontinued for all children of HIV-positive mothers 2. Always prohibit exclusive or mixed breastfeeding in HIV-positive mothers, as well as feeding by wet nurses and milk from human milk banks.	The provision of formula milk until 5 mo and 29 d of age. Starting at 6 mo, infants should be enrolled to the National Complementary feeding program. Pharmacological+F188 suppression of milk production	Ministry of Health	
6. Costa Rica [73,74]	2014–2019	National Standard	Ministry of Health	Mothers should be informed that HIV can be transmitted via breast milk, and breastfeeding should be discontinued to prevent this risk.	The mother will receive a monthly supply of cow's milk-based formula.	Ministry of Health	
7. Dominican Republic [75]	2013	Guideline	Ministry of Public Health; Vice-Ministry of Public Health; General Directorate for the Control of Sexually Transmitted Infections and AIDS; Comprehensive Care Coordination Unit.	To minimize the risk of HIV transmission to children, it is recommended to reinforce the strategy of suppressing breastfeeding in the children of women with HIV.	Not specified in the Guideline	Ministry of Public Health; General Directorate for the Control of Sexually Transmitted Infections and AIDS; Comprehensive Care Coordination Unit.	Breastfeeding is not recommended, but no guidance is provided on infant feeding support.
8. Ecuador [76]	2019	Guidelines	Ministry of Public Health/National Directorate of Prevention and Control Strategies/HIV Strategy National Directorate of Standardization Ecuadorian Society of Infectiology	1. Breastfeeding contraindicated 2. “Discontinue breastfeeding in a serodiscordant woman who is taking PrEP”	Administer 1 mg of cabergoline as a single oral dose to all women on the first postpartum day for lactation suppression, unless there is an obstetric contraindication.	Ministry of Health	The document includes a note stating: "These recommendations are general in nature and do not define a single procedural or therapeutic course of action but rather serve as evidence-based guidance for it.” [Not mandatory]
9. El Salvador [77,78]	1. 2001 2. 2003	1. Decree 2. Guideline	1 Legislative Assembly of the Republic of El Salvador 2. Ministry of Public Health and Social Assistance of El Salvador, in cooperation with UNICEF and UNAIDS.	1. Breastfeeding is contraindicated for newborns from mothers living with HIV (Guideline and Decree) 2. Nutritional counseling should support informed decision-making on infant feeding per WHO, UNAIDS, and UNICEF recommendations.	1. Guideline: a) Women are counseled during prenatal care on reasons to avoid breastfeeding and on replacement feeding, which must be AFASS. b) During delivery and postpartum: Support is provided for breastfeeding cessation and replacement feeding. If delivery occurs in a facility, full replacement feeding is provided for ≥1 y. 2. Decree: The Ministry of Health, with partners (public or private institutions or national or international organizations), ensures the provision and free distribution of breastmilk substitutes through the public health system.	1. Ministry of Public Health and Social Assistance, with the advice of the National Commission Against AIDS (CONASIDA). 2. Guideline: Coordinator of the multidisciplinary team in each health facility. Ministry of Health.	The strategic plan refers to some indicators for screening and treatment monitoring but does not include any guidelines. It also makes no mention of infant feeding recommendations for newborns of mothers living with HIV.
10. Guatemala [79,80]	2008	Guideline and Specific action plan (2011–2015)	Guatemala Government, Ministry of Public Health: Sexual and Reproductive Health Counseling Unit, STI, HIV, and AIDS and CONASIDA	1. When a mother is living with HIV, the guidelines state that if infant formula is AFASS1, breastfeeding should be avoided. 2. If formula feeding is not possible, then exclusive breastfeeding is suggested until 6 mo of age, and it should be discontinued when replacement feeding becomes feasible. 3. The counseling should inform the mother/caregiver about the risks and benefits of 2 of the 5 infant feeding options in the context of HIV recommended by the WHO/UNICEF/USAID foundational document, which are: a) infant formula; b) exclusive breastfeeding.	The provision of formula milk must be ensured.	Ministry of Public Health	1. Based on: Herramientas de consejería en VIH y alimentación infantil, OMS/UNICEF/USAID (2006). Ginebra, Suiza. 2. In Guatemala, after providing all the risk and benefit of two infant feeding options, a counselor does not make the decision for the HIV-positive mother, nor does he or she favor a particular infant feeding method. Whatever the mother’s decision may be—and if it has been based on balanced counseling—it should be supported to ensure she implements it safely.
11. Honduras [81,82]	2008/2021	Guidelines/Handbook	Ministry of Health	1. It is recommended to feed infants exposed to HIV at birth with breastmilk substitutes. It must be ensured that the formula is AFASS1. 2. Formula feeding should be maintained until 6 mo of age and then supplemented with age-appropriate nutritious foods. 3. If, after counseling, the mother decides to breastfeed, it is mandatory to reinforce adherence to ART, maintain undetectable viral loads, and avoid mixed feeding. 4. Counseling should aim to raise the mother's awareness about the risks of HIV transmission through breastfeeding and/or mixed feeding, providing her with the knowledge and tools necessary to make the best decisions to prevent transmission.	The government provides infant formula, ensuring it meets AFASS criteria.	Ministry of Health	
12. Mexico [84,85]	2020–2024	Specific Action Plan	Ministry of Health	All children of women living with HIV, regardless of the prophylaxis they received or the type of maternal ARV regimen, must be fed with formula, ensuring the AFASS1 criteria are met.	Institutions must ensure the provision of formula milk from the immediate postpartum period and for at least the first 6 mo of life.	Ministry of Health/CONASIDA	
13. Nicaragua [86,87]	2008	Norms and Specific Action Plan	Ministry of Health/UNICEF	1. Avoid breastfeeding when possible, opting for infant formula if it is AFASS1. 2. When AFASS1 conditions cannot be ensured, exclusive breastfeeding should be considered until the conditions that meet AFASS criteria are achieved (or ≤4 mo) 3. Counseling and support for mothers to ensure proper feeding, including: a) guidance on feeding alternatives and methods to suppress breast milk production when needed; b) hygienic conditions to minimize health risks, with access to safe water and sanitation; c) monitoring and follow-up for at least the first 2 y of the child's life to ensure proper feeding and HIV transmission.	Not specified.	Ministry of Health (CONISIDA)	
14. Panama [88,89]	2006	Norm	PAHO, INCAP, National Program of STIs/HIV/AIDS and Ministry of Health of Panama	Breastfeeding is absolutely contraindicated for mothers living with HIV, and the use of infant formula is recommended.	Instructions for the administration of appropriate infant formula should be provided. In cases of poverty or extreme poverty, the provision of infant formula will be coordinated.	Ministry of Health	All HIV tests, as well as CD4 and viral load tests, must be provided free of charge to pregnant women attending consultations in the public health system and the Social Security Fund. This ensures access and affordability, aiming to reduce the risk of mother-to-child transmission across all regions of the country.
15. Paraguay [90]	2025	Guideline	The Ministry of Health and Social Welfare, General Directorate of Health Surveillance Paraguay, PRONASIDA Paraguay	Breastfeeding is contraindicated in all cases.	-Infant formula is recommended from birth to 12 mo, regardless of maternal antiretroviral therapy or infant prophylaxis. The Ministry of Health and Social Welfare provides free formula ≤6 mo of age. - Medications such as cabergoline should be administered immediately after delivery to suppress breastfeeding.	The Ministry of Health and Social Welfare	When a woman tests positive on a rapid HIV test during labor, delivery, or postpartum, an appropriate regimen of ART drugs should be started immediately for both the mother and the newborn, and the mother should not breastfeed while awaiting the results of the confirmatory HIV test.
16. Peru [91,92]	1. 2015 2. 2019 3. 2024	Technical law resolution	Ministry of Health of Peru	Breastfeeding should be avoided to reduce infection risk.	Artificial feeding must be provided free of charge through the Comprehensive Health Insurance (SIS).	Ministry of Health	
17. Trinidad and Tobago [93,94]	1. 2010 2. 2021	1 Programm 2. Guideline	Ministry of Health	HIV-positive mothers who choose to breastfeed must be closely monitored, adhere strictly to antiretroviral therapy, and follow all recommendations by their local health care provider (2021).	Not specified.	Ministry of Health	Children born to HIV positive mothers will be tested for HIV at 6 wk after birth by DNA PCR and at 18 mo of age by HIV antibody testing.
18. Uruguay [95,96]	2017– 2013	Norm and Strategic Action Plan	Ministry of Health	In all cases Breastfeeding contraindicated	Ensure the provision of industrially prepared infant formula for girls and boys, children of HIV+ women, ≤6 mo of age by the institution responsible for their care. If possible, the use of infant formula will be promoted until 12 mo of age.	Ministry of Health	
19. Venezuela [97,98]	2012–2013	1. National Strategic Plan 2012–2016 2. Official Standard for Comprehensive Care in Sexual and Reproductive Health	Ministry of People´s Power of Health	Breastfeeding is contraindicated for mothers living with HIV due to the increased risk of vertical transmission	Follow-up on newborn from mothers living with HIV receiving breastmilk substitutes.	National Program for Sexual and Reproductive Health of the Ministry of People´s Power of Health	The strategic plan includes only information on the indicators to be used for monitoring its implementation. It is assumed that the related actions are implicitly considered within the planned activities

Abbreviations: AFASS, acceptable, feasible, affordable, sustainable, and safe; ART, antiretroviral treatment; INCAP, Instituto de Nutrición de Centro América y Panamá; PAHO, Pan American Health Organization; PrEP, pre-exposure prophylaxis; PRONASIDA, Programa Nacional de Control de Sida; STI, sexually transmitted infection.

a While this article was in press, Argentina’s Ministry of Health released a technical document (67), indicating that breastfeeding is not generally recommended, but that an individualized approach for supporting breastfeeding is possible if the individual wishing to breastfeed has sustained viral suppression beginning before pregnancy and maintained throughout gestation.

All guidelines were based on the 1998 [25] and 2010 [40] WHO infant feeding guidelines for WLHIV on HIV. Other than Guatemala [79,80], Honduras [[81], [82], [83]], Nicaragua [86,87], and Trinidad and Tobago [94] countries recommend HIV-positive mothers to avoid breastfeeding and instead provide infant formula. El Salvador [77,78], Guatemala, Honduras, and Nicaragua emphasize the importance of ensuring that AFASS is made available; otherwise, they recommend exclusive breastfeeding for the first 6 mo. In Guatemala, mothers were given the option to choose after being fully informed about the risks and benefits of different feeding methods. Mexico [84,85] also acknowledged the AFASS criteria, but its current normative standards and recommendations prioritize providing infant formula to avoid breastfeeding. Meanwhile, the latest technical recommendation from Argentina [67] and the guideline from Trinidad and Tobago (2021) [94] stipulated that WLHIV who choose to breastfeed must be closely monitored, adhere strictly to ART, and follow all recommendations from their local health care provider.

Support for mothers and their families varied across countries, but most addressed infant formula provision, as well as lactation suppressors, and counseling. Argentina [66], Belize [68], Brazil [71], Chile [72], Costa Rica [73,74], El Salvador [77,78], Guatemala [79,80], Mexico [84,85], Paraguay [90], Perú [91,92], and Uruguay [95,96] prioritized providing infant formula, with Panama [88,89] emphasizing its need in low-income households. The stipulated duration for providing breastmilk substitutes, when mentioned, ranged aged from 6 to 12 mo, although not all countries specified a time frame in their guidelines. Guatemala [79,80], Honduras [81,82], and Nicaragua [86,87] guidelines emphasized offering proper counseling for informed decision making. In Brazil [71] and Chile [72], the recommendations are more specific, including contraindicating mixed feeding, wet nurses, human milk banks, and the use of pasteurized human milk at home. Among the documents reviewed, Argentina [67] and Trinidad and Tobago were the only countries where a partial change in national policy was identified. While in Trinidad and Tobago the 2010 legal framework [93] stated that breastfeeding was not recommended for infants born to WLHIV, as previously mentioned, the current guideline allows WLHIV to breastfeed if they choose to do so, provided they are strictly monitored. A just released techical documentt from Argentina's Ministry of Health released a technical [67], indicating that breastfeeding is not generally recommended, but that an individualized approach for supporting breastfeeding is possible if the individual wishing to breastfeed has sustained viral suppression beginning before pregnancy and maintained throughout gestation.

Additional strategies across countries to reduce MTCT include universal access to HIV screening. Most countries recommend multiple HIV screenings during each pregnancy trimester and initiating ART for WLHIV as early as possible during pregnancy. Although ART regimens vary across countries, they typically recommend triple ART during pregnancy, and some but not all [83] also during the postpartum period (Supplemental Table 1).

Guatemala [79,80] and Honduras [[81], [82], [83]] highlight the importance of providing WLHIV information on the risk of MTCT through breastfeeding and mixed feeding to support informed decision making. Nicaragua goes further by including hygiene information to minimize health risks, emphasizing access to safe water and sanitation [86,87], and endorsing infant feeding monitoring for at least the first 2 y after birth.

The legal framework for infant feeding recommendations for WLHIV was available in the countries for which guidelines and standards were identified. This was reflected in regulations or even specific action plans, especially in Belize, Chile, Costa Rica, Guatemala, Mexico, Nicaragua, Panama, Uruguay, and Venezuela (Table 3 and Supplemental Table 1).

The national regulations in several countries were aligned with the 2016 WHO guideline including: *1*) ensuring that WLHIV receive the necessary care and treatment, including the provision of lifelong ART. *2*) Providing counseling for WLHIV on the risks and benefits of breastfeeding and other feeding options. *3*) Ensuring permanent access to ART while providing information on the importance of proper adherence to treatment. This last recommendation was included in the regulations of Honduras [82,83].

Guatemala’s infant feeding regulations and guidelines among WLHIV included the 2011–2015 Specific Action Plan [80], and the infant feeding guidance documents for WLHIV [66]. Guatemala is the only country that incorporated a gender perspective in its Specific Action Plan. This plan also outlines Guatemala’s legal framework, including Decree 27-2000, entitled the General Law for the Combat of HIV and AIDS, and for the Promotion, Protection, and Defense of Human Rights in the Context of HIV/AIDS [99]. This legislation enables the implementation of essential mechanisms for education, prevention, epidemiological surveillance, research, care, and follow-up related to sexually transmitted infections, HIV, and AIDS, while also ensuring the respect, promotion, protection, and defense of the human rights of affected individuals.

Guatemala’s regulatory framework on infant feeding recommendations promotes shared informed decision-making on safe breastfeeding among WLVIH. It also endorses counseling on sexually transmitted infections, HIV, and AIDS using an evidence-based toolkit [79] (Supplemental Table 1).

In Mexico, the Ministry of Health National Center for Prevention and Control of HIV and AIDS (CENSIDA) develops and implements policies, programs, and research initiatives focused on HIV prevention, diagnosis, treatment, and care [100]. A key informant from CENSIDA reported that a process to update the infant feeding guidelines for WLHIV is in progress, and will likely consider the social, cultural, and economic contexts where the program implementation will take place, and the need for highly trained personnel to implement the programs.

Adherence to HAART medication is the backbone of the updated infant feeding-WLHIV guidance, but implementing it in LAC will be challenging in countries deciding to update their guidance. Starting with ART adherence, A meta-analysis of 53 studies involving 22,603 people living with HIV across 25 LAC countries reported an overall adherence rate of 70% [101]. In Latin America, HAART access among pregnant WLHIV is far from universal, with about one-third not having it, and among those who have it, adherence is suboptimal as reflected by relatively low viral load suppression [102,103]. Factors leading to poor adherence may be setting-specific, including psychosocial factors (such as multiple co-occurring psychosocial health problems [104], economic and social barriers [104], and food insecurity [105], among others). Furthermore, viral load testing in the region decreased from 82% in 2017 to 73% in 2022 [103].

## Discussion

The evidence reviewed in this perspective clearly justifies the decision that several high-income countries have made to update their guidelines to support safe breastfeeding among WLHIV who wish to do so. It also justifies the decision that several LAC countries have made to consider updating their official guidelines.

LAC countries have not updated their breastfeeding-HIV guidelines yet, and only Argentina [67] and the Caribbean nation of Trinidad and Tobago have partially done so. Therefore there was no implementation experience with the most updated infant feeding guidelines for WLHIV to review for this article. Moving forward, there are many implementation considerations that LAC countries will need to address across layers of the socio-ecological model to empower WLHIV who wish to breastfeed to be able to do so safely. First, from a policy perspective, there will need to be universal access for all WLHIV to *1*) timely and universal prenatal HIV testing, *2*) timely initiation and maintenance of lifelong HAART, *3*) effective tracking of viral loads, and *4*) highly qualified breastfeeding support. Second, the offering and integration of these services will require strong intersectoral systems' coordination and the efficient flow of financial, human, and medical resources as well as physical infrastructure. Third, at the community level, it will be key for WLHIV to have access to culturally sensitive, unbiased, and nonstigmatizing person-centered services designed to maximize compliance by providing agency to all WLHIV breastfeeding their children to be able to adhere to recommended best practices. Indeed, qualitative research conducted in high-income countries with WLHIV has identified the need for a more patient-centered approach and infant feeding shared decision-making process [47]. The corresponding guidelines recommend that after presenting evidence about the risks and benefits of breastfeeding in the context of MTCT, the beliefs and values of the mothers are considered to reach an informed shared decision [47]. Fourth, additional implementation challenges at the community level will involve allowing WLHIV to choose the best locations and timing of services to meet them where they are. Fifth, at the policy level, it will be key to update the legal framework to support WLHIV to properly manage their condition while promoting, protecting, and defending their human rights.

At the interpersonal level, it will be key for family and friends supporting the WLHIV to attend the education and counseling sessions with the WLHIV to understand how best to support them. In addition, WLHIV will need to be provided with access to community health workers to support them throughout their MTCT prevention journey, including support with the social determinants of health needs such as food, transportation, and housing.

Finally, at the individual level, it will be necessary for all WLHIV to receive empathetic psycho emotional and social support in addition to the counseling and education involved with the successful self-management of their HIV infection and MTCT prevention. WLHIV will need to have access to highly qualified breastfeeding counseling from individuals who have expertise in how to prevent, identify on time, manage, and monitor conditions that may increase viral load and hence the risk of MTCT, including bleeding nipples, engorgement, and mastitis, among others. In addition to providing breastfeeding counseling, the importance of adhering to HAART will need to be reinforced.

### What process can be followed to lead to policy change

In the Americas, the United States and Canada have fully updated their infant feeding guidelines for WLHIV, whereas in the Latin American region, countries are moving in that direction [16,[106], [107], [108]]. Moving forward, it is key to invest in implementation science research to develop cost-effective scaling-up pathways that are adequate to the diverse contexts where the MTCT prevention program among WLHIV who breastfeed takes place. This area of work can benefit from equivalent work done in the area of breastfeeding [109,110]. It is also important to conduct further research to settle the question of whether mixed feeding (breastfeeding and formula feeding) increases the risk of MTCT or not among WLHIV who are virally suppressed.

In conclusion, this perspective paper documents that the risk of MTCT is exceedingly low when WLHIV receives timely HAART since early pregnancy, has undetectable plasma viral loads, and breastfeeds exclusively during the first 6 mo after giving birth. Hence, it is justifiable that many high-income countries where breastfeeding was previously categorically contraindicated among WLHIV have revised their guidelines, and Latin American countries are considering doing so, to support WLHIV who wish to breastfeed to be able to do so safely.

Implementation research is needed on the ground to ensure that the updated guidelines can be effectively translated into cost-effective, sustainable, large-scale programs across the globe. Research on the effectiveness of HAART at reducing HIV MTCT risk among WLHIV who mix feed during the first 6 mo of life is needed across settings. This research needs to specifically address whether there are national evidence-based policies or programs on the ground or if they need to be developed to ensuring widespread and early HIV screening for pregnant women, and if there are barriers such as limited access to healthcare facilities, stigma, or lack of awareness. Likewise, this future work must document in detail if lifelong HAART is readily available for WLHIV in the countries of interest and if there are challenges related to HIV testing, counseling, drug supply and access, affordability, adherence, or healthcare support as well as to qualified breastfeeding counseling services. Lastly, research is needed to further understand the risk of MTCT among women with bleeding nipples, engorgement, or mastitis who are adhering to HAART and have undetectable viral load levels.

## Author contributions

The authors’ contributions were as follows – RP-E, SHC: responsible for the design of the study; RP-E: drafted the first version of the paper; and all authors: cowrote the paper and read and approved the final version of the manuscript.

## Funding

The authors reported no funding received for this study.

## Conflict of interest

The authors report no conflicts of interest.
