# Supplementary material for: Perspective: Infant Feeding Policies among Women Living with HIV in Latin America and the Caribbean: Should They Be Updated?
Source: Adv Nutr. 2025 Jun 24;17(2):100469. doi: 10.1016/j.advnut.2025.100469 (PMC12919343; doi:10.1016/j.advnut.2025.100469)
Supplement: multimedia component 1 [file mmc1.docx]

**Infant feeding policies among women living with HIV in the Americas: Should they be updated?**

Rafael Pérez-Escamilla, Sonia Hernández Cordero, and Tarini Gupta

**Supplementary Table 1.** Strategies to decrease the vertical transmission of VIH in the Latin American region in the last 20 years. ^1,2^

| Country (Year) | Type of document | Issuing organization | Infant Feeding Recommendation | Infant feeding support | Early HIV diagnosis | Antiretroviral for pregnant women treatment | Antiretroviral for women in the postpartum | Neonatal prophylactic treatment | In charge of its implementation and oversight | Evolution on the recommendation | Reference | Other legal documents or guidelines | Additional notes |
| --- | --- | --- | --- | --- | --- | --- | --- | --- | --- | --- | --- | --- | --- |
| Argentina (2016) | Guidelines | Direction of HIV/AIDS and STIs, Ministry of Health of the Nation, UNICEF | **In all cases  Breastfeeding contraindicated** | The provision of formula milk must be ensured, provided by the Directorate of HIV and STIs of the Ministry of Health of the Nation, from the start for all newborns of mothers with HIV, as well as coverage with lactation inhibitors. | Universal access and offering of the HIV test for pregnant women | Pregnant women should initiate antiretroviral therapy (ART) (or continue is already receiving it) as soon as possible after diagnosis confirmation. | In all cases, indicate the **intrapartum** component of intravenous AZT (zidovudine). /Continuation of antiretroviral therapy in women after childbirth **is not Included** in the document. | All neonates born to mothers with HIV must receive AZT (zidovudine) syrup as soon as possible after birth, ideally starting within 6 hours of life, for a duration of 4 to 6 weeks | Directorate of HIV and STIs of the Ministry of Health of the Nation |  | Dirección de Sida y ETS, Ministerio de Salud de la Nación. Argentina, 2016. Ministerio de Salud de la Nación, Argentina; 2016 | Algoritmos de diagnóstico y tratamiento para el control de las infecciones perinatales por VIH, sífilis, hepatitis B y Chagas Iniciativa ETMI-PLUS. Ministerio de Salud República de Argentina. 2024 [Diagnosis and treatment algorithms for the control of perinatal infections by HIV, syphilis, hepatitis B, and Chagas. ETMI-PLUS Initiative. Ministry of Health, Republic of Argentina. 2024] | Continuation of antiretroviral therapy in women after childbirth is not mentioned in the document. The reason is that the guidelines focus solely on the prevention of HIV transmission to the infant. |
| Belize  (2012-2016) | National Strategic Plan | Ministry of Health | The National Strategic Plan does not explicitly contraindicate breastfeeding **but recommends providing milk substitutes to infants born to HIV-positive mothers.** | The Ministry of Health provides free replacement feeding for the first 10 months to children born to HIV-positive mothers. | HIV testing at first antenatal care visit and at 32 weeks if negative. HIV-positive women referred for treatment and CD4 testing every trhee months. | Provision of prophylaxis to pregnant women at 14 weeks of gestation. | Not specified in the Strategic plan | Infants exposed to HIV receive Zidovudine and Trimethoprim/ Sulfamethoxazole for the first six weeks after birth. | Ministry of Health; National AIDS Commission, National AIDS Commission. | National AIDS Task Force (1996) and subsequently the National AIDS Commission (2003); the adoption of the National HIV Policy (2006), the National HIV/AIDS Workplace Policy (2006), the National Strategic Plan (NSP) for 2006-2011 and the National Monitoring and Evaluation Plan of the NSP 2006 – 2011 (2008) | National AIDS Commission. (2012). Getting to Zero - Belize HIV strategic plan 2012–2016. Belmopan, Belize: National AIDS Commission. | Gender-based Analysis of HIV in Belize (2010); National HIV/AIDS Epidemiologic Profile 2003–2007 | Postpartum antiretrovirals for women not mentioned. |
| Bolivia  (1. 2007; 2. 2017) | 1. Law; 2. Guideline for antiretroviral therapy in children | 1. Honorable National Congress of Bolivia; 2. Ministry of Health of the Plurinational State of Bolivia | **HIV-Positive women advised not to breastfeed (Guideline)** | Not specified in law or guideline. | Voluntary and confidential HIV testing promoted for all pregnant women. | HIV-positive pregnant women entitled to comprehensive care: counseling, scheduled C-section, and antriretroviral treatment to prevent vertical transmission. | Not specified in the law. | Newborns of HIV-positive mothers should be delivered by c-section, receive Zidovudine prophylaxis for 4-6 weeks, and undergo virological testing to confirm or rule out HIV. | The National Sexual Transmitted Infections (STI)/HIV/AIDS Program of the Ministry of Health |  | 1) Bolivia: Law for the Prevention of HIV-AIDS, Protection of Human Rights and Comprehensive Multidisciplinary Assistance for People Living with HIV-AIDS, August 8, 2007. 2) Ministry of Health of Bolivia: Guideline for antiretroviral therapy in Children. La Paz, Bolivia, 2017 |  | Newborns of mothers with HIV must undergo an HIV diagnostic test. Children born to mothers living with HIV-AIDS have the right to receive specialized pediatric services, including access to antiretroviral medications. |
| Brazil (2020) | Guideline | Ministry of Health of Brazil, Secretariat of Health Surveillance, Department of Chronic Conditions and Sexually Transmitted Infections | **a) The mother should be advised to replace breast milk with infant formula until the child reaches 6 months of age.** b) Cross-nursing (feeding the child by another wet nurse) and the use of pasteurized human milk at home are strictly contraindicated. | The government provides free infant formula for children born to HIV-positive mothers. | HIV testing is recommended for all pregnant women in prenatal care, with retesting in the third trimester. | Immediate initiation of antiretroviral therapy (ART) for all pregnant women diagnosed with HIV. | Continuation of ART postpartum, as part of lifelong treatment for women living with HIV. | Newborns exposed to HIV receive prophylactic antiretroviral treatment | The Brazilian Ministry of Health, through the Secretariat of Health Surveillance, in coordination with state and municipal health departments |  | Brasil. Ministerio de Salud. Portaria SCTIE/MS nº 55, de 11 de noviembre de 2020: Aprova o Protocolo Clínico e Diretrizes Terapêuticas para Prevenção da Transmissão Vertical do HIV, Sífilis e Hepatites Virais. Secretaría de Ciencia, Tecnología, Innovación e Insumos Estratégicos [Internet]. Brasília (DF): Ministerio de Salud; 2020 [citado el día mes año]. Available at: https://www.gov.br/aids/pt-br/central-de-conteudo/pcdts | Brasil. Lei nº 9.313, de 13 de novembro de 1996. Dispõe sobre a distribuição gratuita de medicamentos aos portadores do HIV e doentes de AIDS [Internet]. Brasília (DF): Presidência da República; 1996 [Accessed 14 February, 2025. Available at:: https://www.planalto.gov.br/ccivil_03/leis/l9313.htm | In Brazil, there has been a law since 1996 that stipulates, 'People living with HIV (human immunodeficiency virus) and those with AIDS (Acquired Immunodeficiency Syndrome) will receive, free of charge, all necessary medication for their treatment from the Unified Health System. |
| Chile (2012) | National standard | Public Health Undersecretariat, Ministry of Health | 1) **Breastfeeding must be discontinued** for all children of HIV-positive mothers 2) Always prohibit exclusive or mixed breastfeeding in HIV-positive mothers, as well as feeding by wet nurses and milk from human milk banks | The provision of formula milk until 5 months and 29 days of age. Starting at 6 months, infants should be enrolled to the National Complementary feeding program. /Pharmacological+F188 suppression of milk production | Universal access to HIV screening for pregnant women without a known HIV diagnosis during their first prenatal visit | Triple therapy . Initiate ART in pregnant women without prior treatment starting from the 20th week of gestation. ART should be initiated at week 14 if the viral load exceeds 100,000 copies/mL | Use intravenous AZT during labor (during delivery and until cord clamping) | Use of oral AZT suspension in the newborn for 6 weeks, regardless of whether the pregnant women received AZT | Ministry of health | 2005 previous document, but no change in norms | Ministerio de Salud [Del Gobierno de Chile]. Norma General Técnica Nº 0141 del 2012. Norma Conjunta de Prevención de la Transmisión Vertical del VIH y Sífilis. Ministerio de Salud, Chile; 2012 |  |  |
| Costa Rica (2014/ 2019) | National Standard | Ministry of Health | Mothers should be informed that HIV can be transmitted via breast milk, and **breastfeeding should be discontinued to prevent this risk.** | The mother will receive a monthly supply of cow's milk-based formula. | All health services must offer HIV testing to pregnant women during the first visit and last trimester, based on risk assessment and accompanied by counseling on treatment adherence, nutrition, breastfeeding, safe sex, and family planning, prioritizing child´s best interest. | Pregnant women with HIV will be candidates for receiving triple-drug ART during pregnancy.  -Pregnant women with HIV, 12 or more weeks pregnant and without previous ART: start Zidovudine, Lamivudine, and Lopinavir/Ritonavir from week 12 of pregnancy. -In all women receiving ART during pregnancy, increase Lopinavir/Ritonavir to 3 tablets every 12 hours starting in the second trimester. | The Integrated HIV Care Clinic will take the necessary steps to monitor and treat the mother after delivery. - Women with HIV labor who have not previously received ART: Implement the guidelines for labor and for the newborn | Prophylaxis with Zidovudine: the newborn should begin prophylactic treatment with Zidovudine at 8 hours of age and continue for 6 weeks. Zidovudine should be given without mixing it with food, at a dose of 4 mg/kg of body weight/dose every 12 hours. | Ministry of Health |  | 1) Ministerio de Salud. Norma para la Atención Integral de personas con VIH/SIDA No. 38374-S. Ministerio de Salud, República de Costa Rica. 2014. 2) Ministerio de Salud. Norma Nacional para la Atención Integral del VIH en el ámbito de Salud, Artículo 5. Ministerio de Salud, República de Costa Rica. 2019 |  |  |
| Dominican Republic (2013) | Guideline | Ministry of Public Health; Vice-Ministry of Public Health; General Directorate for the Control of Sexually Transmitted Infections and AIDS; Comprehensive Care Coordination Unit | To minimize the risk of HIV transmission to children, it is recommended to reinforce the strategy of suppressing breastfeeding in the children of women with HIV. | Not specified in the Guideline | All pregnant women should be asked to undergo HIV testing during their first prenatal visit. If the test is negative, it should be repeated during the third trimester and at the time of delivery. | **All pregnant women with HIV should start Antiretroviral treatment (ART) as soon as possible during pregnancy**, regardless of CD4, viral load or clinical signs. Recommended regimen: TDF (300 mg/3CT (300 mg)/EFV (600 mg) once daily orally. Alernatives include TDF/4TC/Lopinavir or TDF/3CY/Atazanavir(ritonavir if EFV is not tolerated. | Maintain the pre-delivery medication regimen. | AZT will be administered at a dose of 2 mg/kg of body weight every 6 hours orally for 6 weeks, starting within the first 6–8 hours after delivery. | Ministry of Public Health; General Directorate for the Control of Sexually Transmitted Infections and AIDS; Comprehensive Care Coordination Unit |  | Ministerio de Salud Pública. (2013). Guía Nacional para el Manejo de la Infección por VIH. Santo Domingo, República Dominicana: Ministerio de Salud Pública. ISBN: 978-9945-436-86-0. | 1) Essential prevention and care interventions for adults and adolescents living with HIV in resource-limited settings (2008); Guidance on Provider-Initiated HIV Testing And Counselling in Health Facilities (2007). 2) Drugs for treating pregnant women and preventing HIV infection in infants: recommendations for a public health approach (2010). | Breastfeeding is not recommended, but no guidance is provided on infant feeding support. |
| Ecuador (2019) | Guidelines | Ministry of Public Health/ National Directorate of Prevention and Control Strategies / HIV Strategy National Directorate of Standardization Ecuadorian Society of Infectiology | 1) **Breastfeeding contraindicated** 2) "Discontinue breastfeeding in a serodiscordant woman who is taking PrEP" | Administer 1 mg of cabergoline as a single oral dose to all women on the first postpartum day for lactation suppression, unless there is an obstetric contraindication. | Offer HIV screening to all pregnant women and all women of childbearing age who visit health facilities within the national health system. Conduct HIV screening for pregnant women: • At the first prenatal visit (regardless of gestational age) • During the second trimester of pregnancy • During the third trimester of pregnancy  Perform urgent HIV screening for pregnant women in any obstetric event (delivery, immediate postpartum, late postpartum up to 6 months, abortion, or threatened preterm labor) when screening has not been previously conducted. | Initiate ART in all pregnant women living with HIV as early as possible to prevent mother-to-child transmission, regardless of viral load or CD4+ lymphocyte count. /Prioritize monitoring ART during prenatal check-ups, as well as ensuring adherence in the pregnant woman.  In case HIV positive before pregnancy: Continue ART, adjusting the regimen if necessary (virological failure) according to the resistance test. | Administer ART during both the antepartum and intrapartum periods to the mother to minimize the risk of mother-to-child transmission. [ Initiate ARV prophylaxis with intravenous AZT* in women with a positive or indeterminate diagnosis at the time of delivery, regardless of the ART regimen previously used.] | Administer ART during the postnatal stage to the newborn, to minimize the risk of mother-to-child transmission. | Ministry of Health |  | Ministerio de Salud Pública del Ecuador. Prevención, diagnóstico y tratamiento de la infección por el virus de inmunodeficiencia humana (VIH) en embarazadas, niños, adolescentes y adultos. Guía de Práctica Clínica. Quito: Ministerio de Salud Pública, Dirección Nacional de Normatización; 2019. |  | The document includes a note stating: " These recommendations are general in nature and do not define a single procedural or therapeutic course of action but rather serve as evidence-based guidance for it." [Not mandatory] |
| El Salvador (1. 2001; 2. 2003) | 1. Decree;  2. Guideline | 1.Legislative Assembly of the Republic of El Salvador 2. Ministry of Public Health and Social Assistance of El Salvador, in cooperation with UNICEF and UNAIDS. | 1) Breastfeeding is contraindicated for newborns from mothers living with HIV (Guideline and Decree); 2) Nutritional counseling should support informed decision-making on infant feedingper WHO, UNAIDS, and UNICEF recommendations. | 1. Guideline: a) Women are counseled during prenatal care on reasons to avoid breastfeeding and on replacement feeding, which must be acceptable, feasible, afforable, sustainable, and safe (AFASS). b) During delivery and postpartum: Support is provided for breastfeeding cessation and replacement feeding. If delivery occurs in a facility, full replacement feeding is provided for at least one year. 2. Decree: The Ministry of Health, with partners (public or private institutions or national or international organizations), ensures the provision and free distribution of breastmilk substitutes through the public health system. | All pregnant women are tested. | Not specified in the Decree or the Guideline | -Elective cesarean at 37–38 weeks performed by the most qualified personnel. Emergency cesarean if labor begins in latent phase. -ART-completed pregnancy: AZT 2 mg/kg/h IV for 3 h before cesarean; 1 mg/kg/h during delivery. -No ART during pregnancy: Same AZT protocol + Nevirapine 200 mg orally. -Not in labor: From 14 weeks, oral AZT (200 mg TID or 100 mg five times/day), plus IV AZT before and during cesarean/delivery. | Initiate administration of AZT 2 mg/kg every 6 hours for 6 weeks. | 1) Ministry of Public Health and Social Assistance, with the advice of the National Commission Against AIDS (CONASIDA). 2) Guideline: Coordinator of the multidisciplinary team in each health facility. Ministry of Health. | The decree states: The comprehensive HIV/AIDS care policy will be reviewed and updated according to scientific and technological advances. | 1) Asamblea Legislativa de la República de El Salvador. (2001). Decreto Legislativo No. 588: Ley de Prevención y Control de la Infección Provocada por el Virus de Inmunodeficiencia Humana. 2) Ministerio de Salud Pública y Asistencia Social de El Salvador, UNICEF, & ONUSIDA. (2003). Guía para la prevención de la transmisión materno infantil del VIH (PTMI-VIH). El Salvador: Ministerio de Salud Pública y Asistencia Social. | Ministerio de Salud de El Salvador. (2022). Plan estratégico nacional multisectorial de VIH e ITS 2022-2026. El Salvador: Ministerio de Salud | The strategic plan refers to some indicators for screening and treatment monitoring, but does not include any guidelines. It also makes no mention of infant feeding recommendations for newborns of mothers living with HIV |
| Guatemala (2008) | Guidelines and Specific action plan (2011-2015) | Guatemala Government, Ministry of Public Health: Sexual and Reproductive Health Counseling Unit, STI, HIV, and AIDS and CONASIDA | 1) When a **mother is living with HIV**, the guidelines state that **if infant formula is AFASS1, breastfeeding should be avoided.**  2) I**f formula feeding is not possible, then exclusive breastfeeding** is suggested until 6 months of age, and it should be discontinued when replacement feeding becomes feasible.  3) The counseling should inform the mother/caregiver about the risks and benefits of two of the five infant feeding options in the context of HIV recommended by the WHO/UNICEF/USAID foundational document, which are: a) Infant formula; b) Exclusive breastfeeding. | The provision of formula milk must be ensured, | It is encouraged that pregnant women undergo HIV screening prenatal visits. | Administration of AZT to HIV-positive women during pregnancy |  | Proper intake of antiretrovirals (both in the mother and in the newborn) / Ensure preventive measures for perinatal transmission of HIV prophylaxis | Ministry of Public Health | 2011 (new document, but based on 2008 with no changes) | 1) Unidad de Orientación en ITS, VIH y SIDA, Guatemala. Guía para la orientación sobre alimentación de bebés de mamás con VIH. Dirigida a orientadores(as) en ITS, VIH y SIDA. Guatemala; 2008. 2) Gobierno de la República, Ministerio de Salud y CONASIDA. Plan Estratégico Nacional para la Prevención, Atención y Control de ITS, VIH y Sida 2011-2015. Guatemala; 2011. | Guía de atención nutricional en personas con VIH para el tercer nivel de atención Elaborado por la Unidad de Atención Integral del Programa Nacional de Sida Ministerio de Salud Pública y Asistencia Social Departamento de Regulación de los Programas de Atención a las Personas Guatemala, 2011 | 1) Based on: Herramientas de consejería en VIH y alimentación infantil, OMS/UNICEF/USAID (2006). Ginebra, Suiza. 2) In Guatemala, after providing all the risk and benefit of two infant feeding options, **a counselor does not make the decision for the HIV-positive mother, nor does he or she favor a particular infant feeding method. Whatever the mother’s decision may be – and if it has been based on balanced counseling – it should be supported to ensure she implements it safely.** 3) **The strategic plan incorporates a gender perspective.** There is a legal framework in Guatemala: Guatemala has an active legal framework known as Decree 27-2000, the General Law for the Combat of Human Immunodeficiency Virus (HIV) and Acquired Immunodeficiency Syndrome (AIDS), and for the Promotion, Protection, and Defense of Human Rights in the context of HIV/AIDS. This regulation allows for the implementation of necessary mechanisms for education, prevention, epidemiological surveillance, research, care, and follow-up of Sexually Transmitted Infections, Human Immunodeficiency Virus, and Acquired Immunodeficiency Syndrome, as well as ensuring the respect, promotion, protection, and defense of the human rights of individuals affected by these diseases. |
| Honduras  (2008/ 2021) | Guidelines/Handbook | Ministry of Health | 1) **It is recommended to feed infants exposed to HIV at birth with breastmilk substitutes**. It must be ensured that the formula is AFASS1.  2) Formula feeding should be maintained until 6 months of age and then supplemented with age-appropriate nutritious foods.  3) **If, after counseling, the mother decides to breastfeed, it is mandatory to reinforce adherence to ART**, maintain undetectable viral loads, and avoid mixed feeding.  4) Counseling should aim to raise the mother's awareness about the risks of HIV transmission through breastfeeding and/or mixed feeding, providing her with the knowledge and tools necessary to make the best decisions to prevent transmission | The government provides infant formula, ensuring it meets AFASS (Acceptable, Feasible, Affordable, Sustainable, and Safe) criteria | Pregnant women are tested for HIV, and newborns exposed to HIV undergo PCR testing within the first 72 hours of life | Immediate initiation of ART for all pregnant women with HIV, preferably within the first 7 days of diagnosis. | Administer AZT during the surgical procedure | Newborns receive antiretroviral prophylaxis within the first 6 hours of birth, based on risk classification (monotherapy for low risk, combination therapy for high risk). | Ministry of Health | Updated document for HIV vertical transmission 2008, 2021 and 2023. No changes from 2021 to 2023 (no more information added | 1) Secretaría de Salud [del Gobierno de Honduras]. Manual de atención integral a la embarazada para la prevención de la transmisión del VIH de Madre a hijo e hija. SESAL, Tegucigalpa, Honduras; 2008. 2) Secretaría de Salud [del Gobierno de Honduras]. Manual de atención integral del niño y niña con VIH. Tegucigalpa, Honduras; 2021. | Ministerio de Salud [Gobierno de Honduras]. Manual de atención integral a personas adultas y adolescentes con virus de la inmunodeficiencia humana. M06.2017. Rev 03-2023. Tegucigalpa, M.D.C., Honduras; 2023. |  |
| Mexico (2020-2024) | Specific Action Plan | Ministry of Health | **All children of women living with HIV, regardless of the prophylaxis they received or the type of maternal ARV regimen, must be fed with formula,** ensuring the AFASS1 criteria are met | Institutions must ensure the provision of formula milk from the immediate postpartum period and for at least the first six months of life | Yes | Triple therapy during pregnancy^2^, regardless of the CD4 lymphocyte count and HIV viral load (VL) | Antiretroviral therapy after pregnancy resolution, uninterrupted, regardless of the baseline CD4 cells count | Prophylaxis within the first 6-12 hours of life, up to a maximum period of 72 hours | Ministry of Health/CONASIDA | Censida (Centro Nacional para la Prevención y Control del VIH/SIDA- National Center for the Prevention and Control of HIV/AIDS)/ Plan Estratégico Nacional sobre ITS, VIH y SIDA 2006-2010 2/ | 1) Secretaría de Salud. Programa de Acción Específico. VIH y otras ITS. 2020-2024. México; 2020. 2) Censida/Secretaría de Salud. Guía de manejo antirretroviral de las personas con VIH México. México: Censida/Secretaría de Salud; 2021. | NORMA Oficial Mexicana NOM-010-SSA2-2010, Para la prevención y el control de la infección por Virus de la Inmunodeficiencia Humana (2010) [Mexican Official Standard NOM-010-SSA2-2010, for the Prevention and Control of Human Immunodeficiency Virus Infection] |  |
| Nicaragua (2008) | Norms and Specific Action Plan | Ministry of Health/UNICEF | 1) **Avoid breastfeeding when possible, opting for infant formula if it's AFASS1**.  2) When **AFASS1 conditions cannot be ensured, exclusive breastfeeding should be considered** until the conditions that meet AFASS criteria are achieved (or up to 4 months) 3) Counseling and support for mothers to ensure proper feeding, including: a) guidance on feeding alternatives and methods to suppress breast milk production when needed; b) Hygienic conditions to minimize health risks, with access to safe water and sanitation; c) Monitoring and follow-up for at least the first two years of the child's life to ensure proper feeding and HIV transmission. | Not specified | HIV testing: Pregnant women should be tested for HIV and be informed of the results to receive the appropriate treatment. | pregnant women identified as HIV positive are being treated with three protocols, depending on the gestational age at the time of diagnosis, as well as their viral load and CD4 count. The protocols are:  ACTG 076 Protocol, used when the woman is identified in the early weeks of pregnancy. The medication used is Zidovudine. HIVNET 012 Protocol, used when the woman is identified at the time of delivery. The medication used is Nevirapine. Triple Therapy, used when the woman has a high viral load. The medications and regimens used are: • a) Zidovudine + Lamivudine + Nelfinavir • b) Zidovudine + Lamivudine + Nevirapine. | Children born to mothers with HIV should receive prophylaxis with Trimethoprim-Sulfamethoxazole for 2 months after birth. | Follow-up and comprehensive care: Comprehensive follow-up and care for pregnant women with HIV and their children should be ensured, including the performance of proviral DNA tests within the first 12 months of the child’s life. | Ministry of Health (CONISIDA) |  | 1) Ministerio de Salud, Dirección General de Servicios de Salud. Guía de alimentación y nutrición en la atención de personas con VIH y SIDA. Nicaragua; 2008. 2) Comisión Nicaragüense del SIDA. Plan Estratégico Nacional de ITS, VIH y Sida 2011-2015. Nicaragua; 2011. |  |  |
| Panama (2006) | Norm | Panamerican Health Organization (PAHO), INCAP, National Program of STIs/HIV/AIDS and Ministry of Health of Panama | **Breastfeeding is absolutely contraindicated** for mothers with HIV, and the use of infant formula is recommended. | Instructions for the administration of appropriate infant formula should be provided. In cases of poverty or extreme poverty, the provision of infant formula will be coordinated | Every pregnant woman should undergo testing for the early detection of HIV infection. Every pregnant woman who tests negative in the first half of pregnancy should undergo a second test in the second half of pregnancy to confirm her status after the immunological window period | Every pregnant woman who tests positive for HIV (ELISA and/or rapid test) will be referred to a high-risk clinic or ART clinic for the evaluation of her virological and immunological status (CD4/CD8 and viral load) and the initiation of therapy | All pregnant women with HIV will be given antiretroviral therapy from the onset of labor until the umbilical cord is clamped. | A newborn of a mother with HIV who did not receive ART during pregnancy and labor should be given AZT treatment for six weeks.AZT should be initiated as soon as possible, between 6 and 12 hours after birth.It is not clearly established in the guidelines whether prophylactic treatment for newborns is only for the children of mothers who did not receive treatment during pregnancy. | Ministry of Health |  | 1) Ministerio de Salud [de Panamá], OPS/OMS. Normas para la atención integral a las personas con VIH. OPS/OMS, 2006. 2) Ministerio de Salud, Panamá. Guía para la Atención Nutricional de las Personas con VIH. Panamá, 2007 |  | All HIV tests, as well as CD4 and viral load tests, must be provided free of charge to pregnant women attending consultations in the public health system and the Social Security Fund. This ensures access and affordability, aiming to reduce the risk of mother-to-child transmission across all regions of the country. |
| Paraguay (2025) | Guidelines | The Ministry of Health and Social Welfare, General Directorate of Health Surveillance [Dirección General de Vigilancia de la Salud (DGVS)] Paraguay, PRONASIDA Paraguay | **Breastfeeding is contraindicated in all cases.** | -Infant formula is recommended from birth to 12 months, regardless of maternal antiretroviral therapy or infant prophylaxis. The Ministry of Health and Social Welfare provides free formula up to 6 months of age. - Medications such as cabergoline should be administered immediately after delivery to suppress breastfeeding. | All pregnant women should be tested as early as possible, ideally once per trimester, and, if not done in the third trimester during delivery - HIV is recommended as standard care for all sexually active women and should be routinely included in preconception care. | Early initiation of ART is recommended for all pregnant women to reduce the risk of vertical transmission  The preferred regimen at any stage of pregnancy is TLD (Tenofovir/Lamivudine/Dolutegravir 300/300/50mg), one table daily. | In the postpartum period, the woman's antiretroviral treatment will be continued in accordance with adult antiretroviral treatment guidelines. | Pregnant women delivering in health facilities should receive a rapid HIV test. If reactive, Zidovudine (AZT)should be administrated, and antiretroviral prophylaxis initiated in the newborn. | The Ministry of Health and Social Welfare |  | The Ministry of Health and Social Welfare, et. al. Guía de Manejo para la Eliminación de la Transmisión Materno Infantil de VIH, Sífilis Congénita, Hepatitis B, Chagas. 2025. | Law No. 3,940/09 stipulates in Article 3: “Competent Authority: The prevention, treatment, and care of HIV and AIDS constitute a State Policy for whose development and strengthening the Ministry of Public Health and Social Welfare is responsible, through the National AIDS/STI Control Program (PRONASIDA) as the governing body and other government institutions, in coordination with and in accordance with the strategic plan of the National Response.” | When a woman tests positive on a rapid HIV test during labor, delivery, or postpartum, an appropriate regimen of ART drugs should be started immediately for both the mother and the newborn, a**nd the mother should not breastfeed while awaiting the results of the confirmatory HIV test.** |
| Peru  (1. 2015; 2. 2019; 3. 2024) | Technical law resolution | Ministry of Health of Peru | **Breastfeeding should be avoided to reduce infection risk.** | Artificial feeding must be provided free of charge through the Comprehensive Health Insurance (SIS). | Not specified | Not specified | Not specified | Not specified | Ministry of Health |  | 1) Guía técnica para la consejería en lactancia materna / Ministerio de Salud. Dirección General de Intervenciones Estratégicas en Salud Pública. Dirección de Promoción de la Salud -- Lima: Ministerio de Salud; 2019. 2) Norma técnica de salud para la atención integral de salud neonatal: NTS No 214-MINSA/DIGIESP-2024 (R.M. No 545-2024/MINSA) / Ministerio de Salud. Dirección General de Intervenciones Estratégicas en Salud Pública. Dirección de Intervenciones por Curso de Vida y Cuidado Integral -- Lima: Ministerio de Salud; 2024. |  |  |
| Trinidad and Tobago  (1. 2010; 2. 2021) | 1. Programme; 2. Guideline | Ministry of Health | HIV-Positive mothers who choose to **breastfeed must be closely monitored, adhere strictly to antiretroviral therapy, and follow all recommendations by their local health care provider (2021).** | Not specified. | All "patients" should be offered HIV counselling and testing upon admission, in accordance with the National Testing Policy and HIV testing algorithm. | All HIV positive women will receive antiretroviral treatment and care in accordance with the HIV/AIDS National Treatment Guidelines. | Not specified | Not specified | Ministry of Health | The 2010 document prohibited breastfeeding in women with HIV. The 2021 document suggests close surveillance and fully compliant with the use of the approved ARVs during breastfeeding in those women with HIV whose want to breastfeed | 1. Ministry of Health. Prevention of Mother to Child Transmission of HIV (PMTCT). Government of Trinidad and Tobago. 2010. 2. Ministry of Health, Government of Trinidad and Tobago. Breastfeeding and Beyond: A Guide to Infant and Child Feeding. 2021 |  | Children born to HIV positive mothers will be tested for HIV at 6 weeks after birth by DNA PCR and at 18 months of age by HIV antibody testing. |
| Uruguay  (2017/ 2013) | Norm and Strategic Action Plan | Ministry of health | **In all cases  Breastfeeding contraindicated** | Ensure the provision of industrially prepared infant formula for girls and boys, children of HIV+ women, up to six months of age by the institution responsible for their care. If possible, the use of infant formula will be promoted until twelve months of age | In 1997 (Decree 295/997), the law established the requirement to offer the HIV test to all pregnant women, contingent upon their informed consent. The HIV test must be offered to all pregnant women during the first prenatal visit, between 18 and 23 weeks of pregnancy, and as part of routine third-trimester care. | For women with a prior or early diagnosis, it is ideal to begin ART (antiretroviral therapy) at around 14 weeks of pregnancy. Triple therapy . Initiate ART in pregnant women without prior treatment ( AZT+3TC+LPV/r [AZT: zidovudine; 3TC: lamivudine; LPV/r: lopinavir/ritonavir] OR AZT+3TC+NVP [NVP: nevirapine] depending CD4 levels). As part of pregnancy counseling, HIV-positive women receive information and reinforcement regarding the importance of adhering to antiretroviral treatment | In all cases, starting from the onset of labor or at least 4 hours before a cesarean section, intravenous AZT should be administered until the umbilical cord is clamped. Cesarean section recommended in women with a detectable or unknown viral load at 36 weeks of gestation or later | Prophylactic treatment for the newborn of an HIV-positive mother should always be administered. If the mother was not properly treated during pregnancy, the treatment will be intensified and given for 15 days | Ministry of Health | 2009 (Norm), updated but with same information | 1) Ministerio de Salud Pública Dirección General de la Salud. GUÍA CLÍNICA PARA LA ELIMINACIÓN DE LA SÍFILIS CONGÉNITA Y TRANSMISIÓN VERTICAL DEL VIH. DPES, Área de Salud Sexual y Reproductiva, Programa Nacional ITS-VIH/Sida, Ministerio de Salud Pública Dirección General de la Salud; 2013. 2) Ministerio de Salud, UNICEF, RUANDI. Norma Nacional de Lactancia. Ministerio de Salud, UNICEF, RUANDI; 2017. | MARCO NORMATIVO EN RELACIÓN AL VIH/SIDA EN URUGUAY . Programa Prioritario ITS/SIDA Dirección General de la Salud Ministerio de Salud Pública . UNFPA, Ministerio de Salud Pública República de Uruguay. Programa Prioritario ITS/SIDA |  |
| Venezuela  (1. 2012; 2. 2013) | 1. National Strategic Plan 2012-2016; 2. Official Standard for Comprehensive Care in Sexual and Reproductive Health | Ministry of People´s Power of Health | **Breastfeeding is contraindicated for HIV-positive mothers due to the increased risk of vertical transmission** | Follow-up on newborn from mothers living with HIV receiving breastmilk substitutes | HIV testing is recommended for all pregnant women during prenatal care and, if status is unknown, during delivery or immediately postpartum. | Initiation of ART in HIV-positive pregnant women is mentioned to prevent vertical transmission; includes immunologic and virologic monitoring. Follow-up on coverage of pregnant women with HIV receiving ART during pregnancy | Not specified | Follow-up on coverage of newborns from HIV-positive mothers receiving prophylaxis from birth to sixth weeks of life (in Strategic plan). Not specified in the Official Standard | National Program for Sexual and Reproductive Health of the Ministry of People´s Power of Health |  | 1) Ministerio del Poder Popular para la Salud. (2012). Plan Estratégico Nacional 2012-2016 para la respuesta al VIH y sida y otras ITS. Caracas, Venezuela. 2) Ministerio del Poder Popular para la Salud. (2013). Norma oficial para la atención integral en salud sexual y reproductiva. Caracas: Ministerio del Poder Popular para la Salud. |  | The strategic plan includes only information on the indicators to be used for monitoring its implementation. It is assumed that the related actions are implicitly considered within the planned activities |

^1^ AFASS: acceptability, feasibility, affordability, sustainability, and safety

^2^ In general the triple Therapy includes: a) A nucleoside reverse transcriptase inhibitor (NRTI), such as tenofovir or lamivudine; b) A second NRTI; and c) A third drug from a different class, such as a protease inhibitor (PI) or an integrase inhibitor (INI).
